# 1-*O*-Acetyl-3,4,6-tri-*O*-benzyl-2-*C*-bromo­methyl-2-de­oxy-α-d-glucopyran­ose

**DOI:** 10.1107/S1600536812048453

**Published:** 2012-12-12

**Authors:** Henok H. Kinfe, Felix L. Makolo, Zanele H. Phasha

**Affiliations:** a Research Center for Synthesis and Catalysis, Department of Chemistry, University of Johannesburg (APK Campus), PO Box 524, Auckland Park, Johannesburg, 2006, South Africa

## Abstract

In the title compound, C_30_H_33_BrO_6_, the pyran­ose ring adopts a chair conformation. Two of the *O*-benzyl phenyl rings lie almost perpendicular to C/C/C/O plane formed by the ring atoms not attached to these *O*-benzyl phenyl rings, and form dihedral angles of 85.1 (2) and 64.6 (2)°, while the third *O*-benzyl phenyl ring is twisted so that it makes a dihedral angle 34.9 (2)° to this C/C/C/O plane. This twist is ascribed to the formation of an *S*(8) loop stabilized by a weak intra­molecular C—H⋯O hydrogen bond.

## Related literature

For background to derivatization of cyclo­propyl carbohydrates, see: Halton & Harvey (2006 ▶); Beyer & Madsen (1998 ▶). For details of the synthesis of the title compound, see: Gammon *et al.* (2007 ▶). For ring puckering analysis, see: Cremer & Pople (1975 ▶).
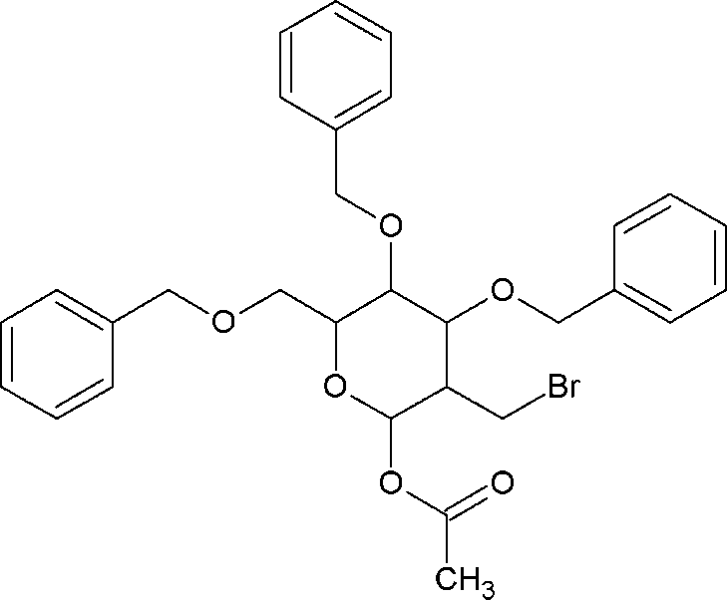


## Experimental

### 

#### Crystal data

C_30_H_33_BrO_6_
*M* *_r_* = 569.47
Orthorhombic, 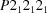

*a* = 5.5097 (4) Å
*b* = 19.9357 (11) Å
*c* = 24.4597 (16) Å
*V* = 2686.6 (3) Å^3^
*Z* = 4
Cu *K*α radiation
μ = 2.43 mm^−1^
*T* = 100 K
0.43 × 0.05 × 0.05 mm

#### Data collection

Bruker APEX DUO 4K-CCD diffractometer
Absorption correction: multi-scan (*SADABS*, Bruker, 2008 ▶) *T*_min_ = 0.613, *T*_max_ = 0.753
51826 measured reflections
4637 independent reflections
4177 reflections with *I* > 2σ(*I*)
*R*_int_ = 0.097

#### Refinement

*R*[*F* ^2^ > 2σ(*F* ^2^)] = 0.041
*wR*(*F* ^2^) = 0.096
*S* = 1.04
4637 reflections
335 parameters
H-atom parameters constrained
Δρ_max_ = 1.04 e Å^−3^
Δρ_min_ = −0.61 e Å^−3^
Absolute structure: Flack (1983 ▶), 1893 Friedel pairs
Flack parameter: −0.01 (2)

### 

Data collection: *APEX2* (Bruker, 2011 ▶); cell refinement: *SAINT* (Bruker, 2008 ▶); data reduction: *SAINT* (Bruker, 2008 ▶); program(s) used to solve structure: *SUPERFLIP* (Palatinus & Chapuis, 2007 ▶); program(s) used to refine structure: *SHELXL97* (Sheldrick, 2008 ▶); molecular graphics: *OLEX2* (Dolomanov *et al.* 2009 ▶); software used to prepare material for publication: *OLEX2*.

## Supplementary Material

Crystal structure: contains datablock(s) global, I. DOI: 10.1107/S1600536812048453/hg5273sup1.cif

Structure factors: contains datablock(s) I. DOI: 10.1107/S1600536812048453/hg5273Isup2.hkl

Additional supplementary materials:  crystallographic information; 3D view; checkCIF report

## Figures and Tables

**Table 1:** Hydrogen-bond geometry (Å, °)

*D*—H⋯*A*	*D*—H	H⋯*A*	*D*⋯*A*	*D*—H⋯*A*
C18—H18⋯O4	0.95	2.85	3.663 (7)	144

**Fig. 1.**
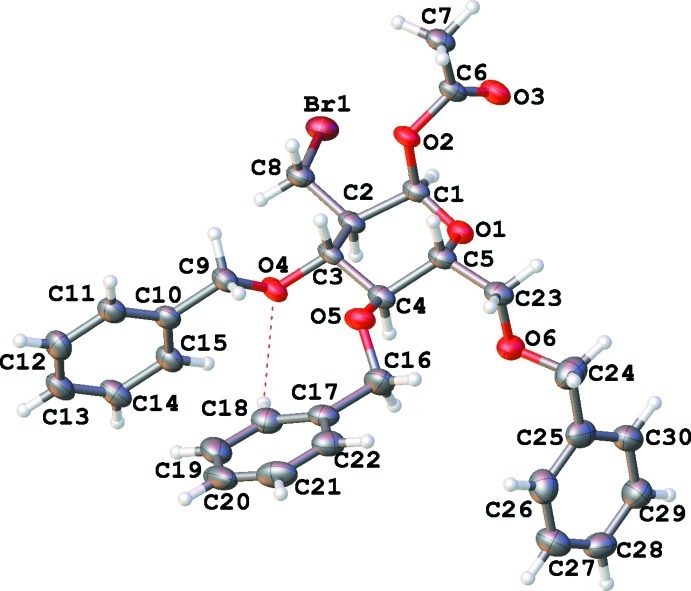
Crystal structure of the title compound with labeling and displacement ellipsoids drawn at the 50% probability level.

## supplementary crystallographic information

### Comment

The derivatization of cyclopropyl carbohydrates remains a relatively small area of research and, of this work, only a small proportion is devoted to reactions of the cyclopropyl moiety (Halton *et al.*, 2006). Nucleophilic attack by a range of alcohols, including monosaccharides afforded 2-C-branched glycosides (Beyer *et al.*, 1998). The compound of interest is a useful glycosyl donor and a precursor for the extension of the C–2 side chain (Gammon *et al.*, 2007).

In the title compound the pyranose ring adopts a chair conformation with ring puckering parameters of q_2_ = 0.0601 Å, q_3_ = 0.5555 Å, Q = 0.5587 Å, θ = 6.17 ° and φ_2_ = 280.0707° (see Cremer & Pople, 1975) (see Figure 1). The C10 – C15 and C25 – C30 rings, lie almost perpendicular to the C1 C3 C4 O1 plane of the pyranose ring, with dihedral angles of 85.1 (2) and 64.6 (2) ° respectively. The C17 – C22 phenyl ring is twisted to an almost parallel position to the C1 C3 C4 O1 plane with a dihedral angle of 34.9 (2)°. The twist is ascribed to the formation of an *S*(8) loop stabilized by weak intramolecular C–H···O hydrogen bond (see Figure 1; Table 1).

### Experimental

The cyclopropane, 3,4,6–Tri–O–benzyl–1,5–anhydro–2–deoxy–1,2– C–methylene–D–glycero–D–gulo–hexitol (120 mg, 0.28 mmol) was suspended in 1:1 mixture of water and 1,4–dioxane (2 mL), N-Bromosuccinamide(NBS) (163 mg, 0.84 mmol) was added, then stirring was allowed overnight at room temperature. The reaction mixture was diluted with 10% aqueous solution of sodium thiosulfate, Na_2_S_2_O_3_, and the aqueous phase extracted with ethyl acetate; the combined organic extracts were dried over MgSO_4_, and concentrated *in vacuo*. The residue was acetylated without purification, acetic anhydride (0.5 ml) in pyridine (1 mL) at room temperature. After 1 h, water was added to the reaction, and the mixture was extracted with dichloromethane. The combined extracts were washed with 1*M* HCl and saturated aqueous NaHCO_3_, dried over MgSO_4_, concentrated under reduced pressure, and purified by flash chromatography to afford the title compound as a white solid.

Analytical data: mp: 115 – 117 °C; ^1^H NMR (CDCl_3_, 400 MHz): σ 7.45 – 7.10 (m, 15H, Aromatic), 6.39 (d, *J* = 2.4Hz, 1H, H-1), 4.92 (d, *J* = 11.2Hz, 1H, C*H_2_*Ph), 4.78 (d, *J* = 10.8Hz, 1H, C*H_2_*Ph), 4.70 – 4.40 (m, 4H, C*H_2_*Ph), 3.90 – 3.55 (m , 6H, H–3, H–4, H–5, H–6_a_, H–6_b_, H–7_a_), 3.12 (t, *J* = 10.6Hz, 1H, H7_b_), 2.45 – 2.25 (m, 1H, H–2), 2.09 (s, 3H, OCOC*H_3_*); ^13^C NMR (CDCl_3_, 100 MHz): σ 168.9 (O*C*OCH_3_), 137.8, 128.6, 128.5, 128.4, 128.0, 128.0, 127.9, 127.8, 127.8, 127.7( Aromatic), 92.1 (C–1), 79.7 (C–3), 78.8 (C–4), 75.4 (C–5), 75.0 (*C*H_2_Ph), 73.6 (*C*H_2_Ph), 73.3 (*C*H_2_Ph), 68.1(C–6), 46.8 (C–2), 29.1(C–7), 20.9 (OCO*C*H_3_).

### Refinement

All hydrogen atoms were positioned in geometrically idealized positions with C–H = 1.00 Å (methine), 0.99 Å (methylene), 0.95 Å aromatic and 0.98 Å (methyl). All hydrogen atoms were allowed to ride on their parent atoms with *U*_iso_(H) = 1.2*U*_eq_, except for the methyl where *U*_iso_(H) = 1.5*U*_eq_ was utilized. The initial positions of methyl hydrogen atoms were located from a Fourier difference map and refined as a fixed rotor.The D enantiomer refined to a final Flack parameter of -0.01 (2). The highest residual electron density of 1.04 e.Å^-3^ is 0.93 Å from Br1.

### Crystal data

**Table:**

C_30_H_33_BrO_6_	*F*(000) = 1184
*M**_r_* = 569.47	*D*_x_ = 1.408 Mg m^−^^3^
Orthorhombic, *P*2_1_2_1_2_1_	Cu *K*α radiation, λ = 1.54178 Å
Hall symbol: P 2ac 2ab	Cell parameters from 9957 reflections
*a* = 5.5097 (4) Å	θ = 4.2–65.2°
*b* = 19.9357 (11) Å	µ = 2.43 mm^−^^1^
*c* = 24.4597 (16) Å	*T* = 100 K
*V* = 2686.6 (3) Å^3^	Needle, colourless
*Z* = 4	0.43 × 0.05 × 0.05 mm

### Data collection

**Table:**

Bruker APEX DUO 4K-CCD diffractometer	4637 independent reflections
Radiation source: fine-focus sealed tube	4177 reflections with *I* > 2σ(*I*)
Incoatec Quazar Multilayer Mirror monochromator	*R*_int_ = 0.097
Detector resolution: 8.4 pixels mm^-1^	θ_max_ = 66.2°, θ_min_ = 4.2°
φ and ω scans	*h* = −6→5
Absorption correction: multi-scan (*SADABS*, Bruker, 2008)	*k* = −23→23
*T*_min_ = 0.613, *T*_max_ = 0.753	*l* = −28→28
51826 measured reflections	

### Refinement

**Table:**

Refinement on *F*^2^	Secondary atom site location: difference Fourier map
Least-squares matrix: full	Hydrogen site location: inferred from neighbouring sites
*R*[*F*^2^ > 2σ(*F*^2^)] = 0.041	H-atom parameters constrained
*wR*(*F*^2^) = 0.096	*w* = 1/[σ^2^(*F*_o_^2^) + (0.0448*P*)^2^ + 1.595*P*] where *P* = (*F*_o_^2^ + 2*F*_c_^2^)/3
*S* = 1.04	(Δ/σ)_max_ = 0.001
4637 reflections	Δρ_max_ = 1.04 e Å^−^^3^
335 parameters	Δρ_min_ = −0.61 e Å^−^^3^
0 restraints	Absolute structure: Flack (1983), 1893 Friedel pairs
Primary atom site location: structure-invariant direct methods	Flack parameter: −0.01 (2)

### Special details

**Table:**

Experimental. Absorption correction: SADABS-2008/1 (Bruker,2008) was used for absorption correction. wR2(int) was 0.1272 before and 0.0933 after correction. The Ratio of minimum to maximum transmission is 0.8137. The λ/2 correction factor is 0.0015.The intensity data was collected on a Bruker Apex DUO 4 K CCD diffractometer using an exposure time of 5 s/frame. A total of 4554 frames were collected with a frame width of 1° covering up to θ = 66.16° with 99.6% completeness accomplished.
Geometry. All s.u.'s (except the s.u. in the dihedral angle between two l.s. planes) are estimated using the full covariance matrix. The cell s.u.'s are taken into account individually in the estimation of s.u.'s in distances, angles and torsion angles; correlations between s.u.'s in cell parameters are only used when they are defined by crystal symmetry. An approximate (isotropic) treatment of cell s.u.'s is used for estimating s.u.'s involving l.s. planes.
Refinement. Refinement of *F*^2^ against ALL reflections. The weighted *R*-factor *wR* and goodness of fit *S* are based on *F*^2^, conventional *R*-factors *R* are based on *F*, with *F* set to zero for negative *F*^2^. The threshold expression of *F*^2^ > 2σ(*F*^2^) is used only for calculating *R*-factors(gt) *etc*. and is not relevant to the choice of reflections for refinement. *R*-factors based on *F*^2^ are statistically about twice as large as those based on *F*, and *R*- factors based on ALL data will be even larger.

### Fractional atomic coordinates and isotropic or equivalent isotropic displacement parameters (Å^2^)

**Table:**

	*x*	*y*	*z*	*U*_iso_*/*U*_eq_	
Br1	1.52718 (7)	0.657227 (17)	0.318194 (16)	0.03952 (13)	
O1	1.1736 (6)	0.61572 (12)	0.48483 (10)	0.0340 (6)	
O2	1.0466 (5)	0.68570 (10)	0.41379 (9)	0.0326 (6)	
O3	1.2526 (6)	0.76628 (11)	0.45934 (11)	0.0400 (7)	
O4	1.0000 (6)	0.48064 (10)	0.36332 (9)	0.0351 (6)	
O5	0.7051 (6)	0.48859 (11)	0.45817 (11)	0.0392 (7)	
O6	1.0735 (6)	0.52549 (12)	0.57192 (10)	0.0396 (7)	
C1	1.2250 (8)	0.63710 (16)	0.43169 (15)	0.0316 (9)	
H1	1.3896	0.6582	0.431	0.038*	
C2	1.2183 (8)	0.57915 (16)	0.39091 (15)	0.0330 (9)	
H2	1.3572	0.5485	0.3992	0.04*	
C3	0.9832 (8)	0.53928 (14)	0.39714 (13)	0.0297 (8)	
H3	0.8448	0.5675	0.384	0.036*	
C4	0.9363 (7)	0.51944 (16)	0.45609 (14)	0.0319 (8)	
H4	1.0638	0.4873	0.4688	0.038*	
C5	0.9433 (8)	0.58369 (16)	0.49090 (14)	0.0338 (9)	
H5	0.8151	0.6149	0.4771	0.041*	
C6	1.0804 (8)	0.74997 (16)	0.43301 (15)	0.0312 (9)	
C7	0.8758 (8)	0.79337 (17)	0.41525 (16)	0.0366 (10)	
H7A	0.9038	0.8394	0.4279	0.055*	
H7B	0.7239	0.7765	0.4309	0.055*	
H7C	0.8646	0.7929	0.3753	0.055*	
C8	1.2419 (8)	0.60206 (17)	0.33175 (15)	0.0375 (10)	
H8A	1.0952	0.6279	0.3217	0.045*	
H8B	1.249	0.562	0.3078	0.045*	
C9	0.7950 (8)	0.46920 (17)	0.33029 (15)	0.0355 (9)	
H9A	0.7645	0.5089	0.3069	0.043*	
H9B	0.6505	0.462	0.3536	0.043*	
C10	0.8379 (8)	0.40787 (17)	0.29466 (15)	0.0325 (9)	
C11	0.6658 (8)	0.39109 (18)	0.25596 (15)	0.0384 (10)	
H11	0.5251	0.4181	0.2515	0.046*	
C12	0.6995 (9)	0.3342 (2)	0.22338 (16)	0.0443 (10)	
H12	0.5808	0.3224	0.1969	0.053*	
C13	0.9042 (9)	0.29499 (18)	0.22953 (17)	0.0436 (11)	
H13	0.9269	0.2565	0.2072	0.052*	
C14	1.0756 (9)	0.31201 (18)	0.26817 (17)	0.0425 (11)	
H14	1.2165	0.2851	0.2726	0.051*	
C15	1.0426 (9)	0.36864 (16)	0.30087 (15)	0.0371 (9)	
H15	1.1612	0.3802	0.3274	0.045*	
C16	0.6728 (10)	0.43619 (19)	0.49772 (17)	0.0467 (11)	
H16A	0.5836	0.4537	0.5298	0.056*	
H16B	0.8329	0.4197	0.5102	0.056*	
C17	0.5316 (9)	0.37961 (16)	0.47184 (15)	0.0367 (9)	
C18	0.6145 (8)	0.35048 (19)	0.42360 (16)	0.0412 (10)	
H18	0.7611	0.3657	0.4073	0.049*	
C19	0.4844 (11)	0.29972 (18)	0.39955 (17)	0.0517 (12)	
H19	0.5387	0.2812	0.3659	0.062*	
C20	0.2765 (11)	0.2752 (2)	0.4234 (2)	0.0604 (15)	
H20	0.1886	0.2398	0.4066	0.072*	
C21	0.1970 (10)	0.3025 (2)	0.4719 (2)	0.0552 (13)	
H21	0.0544	0.2858	0.4889	0.066*	
C22	0.3263 (9)	0.35477 (19)	0.49587 (17)	0.0441 (10)	
H22	0.2712	0.3734	0.5294	0.053*	
C23	0.9028 (8)	0.57286 (17)	0.55147 (15)	0.0371 (10)	
H23A	0.9214	0.616	0.5711	0.045*	
H23B	0.7359	0.5562	0.5577	0.045*	
C24	1.0720 (10)	0.5265 (2)	0.62974 (16)	0.0533 (12)	
H24A	0.9042	0.5199	0.6432	0.064*	
H24B	1.1306	0.5705	0.643	0.064*	
C25	1.2327 (9)	0.4717 (2)	0.65142 (16)	0.0442 (11)	
C26	1.1766 (9)	0.4043 (2)	0.64257 (18)	0.0504 (11)	
H26	1.0354	0.3925	0.6225	0.06*	
C27	1.3272 (9)	0.3549 (2)	0.66308 (18)	0.0511 (11)	
H27	1.288	0.309	0.6572	0.061*	
C28	1.5319 (10)	0.37129 (19)	0.69168 (16)	0.0496 (11)	
H28	1.635	0.3371	0.7056	0.06*	
C29	1.5872 (10)	0.4378 (2)	0.70016 (17)	0.0524 (12)	
H29	1.7305	0.4496	0.7195	0.063*	
C30	1.4362 (9)	0.48721 (18)	0.68078 (17)	0.0470 (10)	
H30	1.4737	0.5329	0.6879	0.056*	

### Atomic displacement parameters (Å^2^)

**Table:**

	*U*^11^	*U*^22^	*U*^33^	*U*^12^	*U*^13^	*U*^23^
Br1	0.0362 (2)	0.02898 (18)	0.0534 (2)	0.00229 (17)	−0.00030 (19)	0.00481 (16)
O1	0.0390 (18)	0.0234 (12)	0.0397 (14)	0.0005 (11)	−0.0060 (13)	−0.0011 (10)
O2	0.0385 (17)	0.0174 (10)	0.0419 (12)	0.0030 (11)	−0.0051 (13)	−0.0026 (9)
O3	0.0414 (19)	0.0239 (12)	0.0546 (16)	−0.0036 (12)	−0.0056 (14)	−0.0026 (11)
O4	0.0393 (17)	0.0219 (10)	0.0442 (12)	0.0038 (12)	−0.0062 (14)	−0.0071 (9)
O5	0.0436 (19)	0.0251 (12)	0.0488 (15)	−0.0033 (12)	−0.0060 (13)	0.0063 (11)
O6	0.050 (2)	0.0314 (13)	0.0374 (13)	0.0091 (12)	0.0003 (13)	0.0005 (10)
C1	0.031 (2)	0.0222 (16)	0.041 (2)	0.0025 (15)	−0.0038 (17)	−0.0011 (14)
C2	0.036 (2)	0.0192 (16)	0.044 (2)	0.0052 (15)	−0.0054 (18)	−0.0032 (14)
C3	0.029 (2)	0.0164 (13)	0.0433 (18)	0.0013 (16)	−0.0066 (19)	−0.0051 (12)
C4	0.030 (2)	0.0226 (16)	0.0433 (19)	0.0013 (15)	−0.0044 (17)	−0.0017 (14)
C5	0.035 (3)	0.0235 (16)	0.0432 (19)	0.0036 (16)	−0.0019 (18)	0.0001 (14)
C6	0.034 (3)	0.0189 (15)	0.0402 (19)	−0.0019 (15)	0.0061 (18)	−0.0009 (14)
C7	0.042 (3)	0.0223 (17)	0.045 (2)	0.0051 (16)	0.0039 (19)	−0.0017 (15)
C8	0.045 (3)	0.0234 (17)	0.044 (2)	0.0029 (17)	−0.0037 (19)	−0.0003 (15)
C9	0.040 (2)	0.0240 (17)	0.043 (2)	0.0009 (16)	−0.0043 (18)	−0.0046 (15)
C10	0.035 (2)	0.0242 (17)	0.0384 (19)	−0.0051 (16)	−0.0012 (18)	0.0012 (15)
C11	0.044 (3)	0.0278 (18)	0.044 (2)	−0.0050 (17)	0.000 (2)	−0.0003 (16)
C12	0.053 (3)	0.036 (2)	0.044 (2)	−0.011 (2)	−0.005 (2)	−0.0057 (17)
C13	0.056 (3)	0.0254 (18)	0.049 (2)	−0.0059 (18)	0.005 (2)	−0.0069 (16)
C14	0.043 (3)	0.0265 (18)	0.058 (2)	−0.0018 (17)	0.003 (2)	−0.0060 (17)
C15	0.039 (3)	0.0280 (16)	0.0443 (19)	−0.0057 (17)	−0.0024 (19)	−0.0044 (14)
C16	0.058 (3)	0.033 (2)	0.049 (2)	−0.010 (2)	−0.005 (2)	0.0031 (18)
C17	0.039 (3)	0.0245 (16)	0.047 (2)	0.0043 (18)	−0.002 (2)	0.0058 (14)
C18	0.040 (3)	0.034 (2)	0.049 (2)	0.0060 (19)	−0.0005 (18)	0.0030 (18)
C19	0.068 (4)	0.0322 (19)	0.055 (2)	0.012 (2)	−0.008 (3)	−0.0039 (17)
C20	0.082 (4)	0.027 (2)	0.072 (3)	−0.007 (2)	−0.031 (3)	0.007 (2)
C21	0.045 (3)	0.042 (2)	0.078 (3)	−0.011 (2)	−0.006 (3)	0.021 (2)
C22	0.047 (3)	0.035 (2)	0.050 (2)	0.0075 (19)	0.004 (2)	0.0066 (18)
C23	0.040 (3)	0.0254 (17)	0.046 (2)	0.0060 (16)	−0.0008 (18)	0.0004 (15)
C24	0.057 (4)	0.060 (3)	0.042 (2)	0.015 (2)	0.001 (2)	0.0043 (19)
C25	0.055 (3)	0.041 (2)	0.036 (2)	0.011 (2)	0.011 (2)	0.0016 (17)
C26	0.045 (3)	0.055 (3)	0.051 (2)	0.004 (2)	−0.006 (2)	−0.009 (2)
C27	0.054 (3)	0.034 (2)	0.065 (3)	−0.001 (2)	0.002 (2)	−0.0014 (19)
C28	0.062 (3)	0.0396 (19)	0.047 (2)	0.010 (2)	0.003 (2)	0.0092 (16)
C29	0.062 (4)	0.047 (2)	0.049 (2)	0.000 (2)	−0.011 (2)	0.0013 (19)
C30	0.062 (3)	0.0329 (18)	0.046 (2)	0.0002 (19)	0.000 (3)	0.0022 (18)

### Geometric parameters (Å, º)

**Table:**

Br1—C8	1.947 (4)	C12—C13	1.381 (6)
O1—C1	1.397 (4)	C13—H13	0.95
O1—C5	1.428 (5)	C13—C14	1.378 (6)
O2—C1	1.448 (4)	C14—H14	0.95
O2—C6	1.377 (4)	C14—C15	1.395 (5)
O3—C6	1.192 (5)	C15—H15	0.95
O4—C3	1.435 (3)	C16—H16A	0.99
O4—C9	1.407 (5)	C16—H16B	0.99
O5—C4	1.415 (5)	C16—C17	1.510 (6)
O5—C16	1.435 (5)	C17—C18	1.392 (6)
O6—C23	1.423 (5)	C17—C22	1.367 (6)
O6—C24	1.414 (5)	C18—H18	0.95
C1—H1	1	C18—C19	1.373 (6)
C1—C2	1.527 (5)	C19—H19	0.95
C2—H2	1	C19—C20	1.375 (8)
C2—C3	1.528 (6)	C20—H20	0.95
C2—C8	1.523 (5)	C20—C21	1.375 (7)
C3—H3	1	C21—H21	0.95
C3—C4	1.517 (5)	C21—C22	1.392 (6)
C4—H4	1	C22—H22	0.95
C4—C5	1.538 (5)	C23—H23A	0.99
C5—H5	1	C23—H23B	0.99
C5—C23	1.514 (5)	C24—H24A	0.99
C6—C7	1.486 (5)	C24—H24B	0.99
C7—H7A	0.98	C24—C25	1.503 (6)
C7—H7B	0.98	C25—C26	1.394 (6)
C7—H7C	0.98	C25—C30	1.367 (7)
C8—H8A	0.99	C26—H26	0.95
C8—H8B	0.99	C26—C27	1.383 (7)
C9—H9A	0.99	C27—H27	0.95
C9—H9B	0.99	C27—C28	1.367 (7)
C9—C10	1.520 (5)	C28—H28	0.95
C10—C11	1.381 (6)	C28—C29	1.377 (6)
C10—C15	1.381 (6)	C29—H29	0.95
C11—H11	0.95	C29—C30	1.373 (6)
C11—C12	1.399 (5)	C30—H30	0.95
C12—H12	0.95		
			
C1—O1—C5	114.4 (3)	C13—C12—H12	119.8
C6—O2—C1	115.3 (3)	C12—C13—H13	120.2
C9—O4—C3	114.3 (3)	C14—C13—C12	119.7 (4)
C4—O5—C16	116.9 (3)	C14—C13—H13	120.2
C24—O6—C23	109.8 (3)	C13—C14—H14	119.9
O1—C1—O2	110.3 (3)	C13—C14—C15	120.2 (4)
O1—C1—H1	109.2	C15—C14—H14	119.9
O1—C1—C2	111.8 (3)	C10—C15—C14	120.1 (4)
O2—C1—H1	109.2	C10—C15—H15	119.9
O2—C1—C2	107.0 (3)	C14—C15—H15	119.9
C2—C1—H1	109.2	O5—C16—H16A	109.9
C1—C2—H2	108.1	O5—C16—H16B	109.9
C1—C2—C3	110.4 (3)	O5—C16—C17	109.0 (3)
C3—C2—H2	108.1	H16A—C16—H16B	108.3
C8—C2—C1	113.1 (3)	C17—C16—H16A	109.9
C8—C2—H2	108.1	C17—C16—H16B	109.9
C8—C2—C3	108.9 (3)	C18—C17—C16	119.9 (4)
O4—C3—C2	108.1 (3)	C22—C17—C16	121.1 (4)
O4—C3—H3	108.8	C22—C17—C18	119.0 (4)
O4—C3—C4	110.3 (2)	C17—C18—H18	120
C2—C3—H3	108.8	C19—C18—C17	119.9 (4)
C4—C3—C2	112.0 (3)	C19—C18—H18	120
C4—C3—H3	108.8	C18—C19—H19	119.5
O5—C4—C3	107.5 (3)	C18—C19—C20	121.0 (4)
O5—C4—H4	110.1	C20—C19—H19	119.5
O5—C4—C5	111.4 (3)	C19—C20—H20	120.3
C3—C4—H4	110.1	C19—C20—C21	119.4 (4)
C3—C4—C5	107.7 (3)	C21—C20—H20	120.3
C5—C4—H4	110.1	C20—C21—H21	120.1
O1—C5—C4	109.7 (3)	C20—C21—C22	119.8 (5)
O1—C5—H5	108.3	C22—C21—H21	120.1
O1—C5—C23	107.3 (3)	C17—C22—C21	120.8 (4)
C4—C5—H5	108.3	C17—C22—H22	119.6
C23—C5—C4	114.8 (3)	C21—C22—H22	119.6
C23—C5—H5	108.3	O6—C23—C5	109.9 (3)
O2—C6—C7	109.8 (3)	O6—C23—H23A	109.7
O3—C6—O2	123.1 (3)	O6—C23—H23B	109.7
O3—C6—C7	127.1 (3)	C5—C23—H23A	109.7
C6—C7—H7A	109.5	C5—C23—H23B	109.7
C6—C7—H7B	109.5	H23A—C23—H23B	108.2
C6—C7—H7C	109.5	O6—C24—H24A	109.7
H7A—C7—H7B	109.5	O6—C24—H24B	109.7
H7A—C7—H7C	109.5	O6—C24—C25	109.8 (3)
H7B—C7—H7C	109.5	H24A—C24—H24B	108.2
Br1—C8—H8A	108.8	C25—C24—H24A	109.7
Br1—C8—H8B	108.8	C25—C24—H24B	109.7
C2—C8—Br1	113.6 (3)	C26—C25—C24	121.0 (5)
C2—C8—H8A	108.8	C30—C25—C24	120.3 (4)
C2—C8—H8B	108.8	C30—C25—C26	118.8 (4)
H8A—C8—H8B	107.7	C25—C26—H26	120.1
O4—C9—H9A	109.8	C27—C26—C25	119.8 (5)
O4—C9—H9B	109.8	C27—C26—H26	120.1
O4—C9—C10	109.6 (3)	C26—C27—H27	119.7
H9A—C9—H9B	108.2	C28—C27—C26	120.6 (4)
C10—C9—H9A	109.8	C28—C27—H27	119.7
C10—C9—H9B	109.8	C27—C28—H28	120.3
C11—C10—C9	118.8 (4)	C27—C28—C29	119.4 (4)
C15—C10—C9	121.3 (3)	C29—C28—H28	120.3
C15—C10—C11	119.9 (3)	C28—C29—H29	119.8
C10—C11—H11	120.1	C30—C29—C28	120.3 (5)
C10—C11—C12	119.7 (4)	C30—C29—H29	119.8
C12—C11—H11	120.1	C25—C30—C29	121.0 (4)
C11—C12—H12	119.8	C25—C30—H30	119.5
C13—C12—C11	120.4 (4)	C29—C30—H30	119.5

### Hydrogen-bond geometry (Å, º)

**Table:**

*D*—H···*A*	*D*—H	H···*A*	*D*···*A*	*D*—H···*A*
C18—H18···O4	0.95	2.85	3.663 (7)	144
